# Impact of hepatic steatosis and MOLLI acquisition parameters on liver T1 estimates

**DOI:** 10.1007/s00261-026-05473-6

**Published:** 2026-04-02

**Authors:** Amol S. Pednekar, Anandh Kilpattu Ramaniharan, Joshua S. Greer, Pradipta Debnath, Andrew T. Trout, Jonathan R. Dillman, Mary Kate Manhard

**Affiliations:** 1 Department of Radiology, Cincinnati Children’s Hospital Medical Center, Cincinnati, USA; 2 Department of Radiology, University of Cincinnati Medical Center, Cincinnati, USA; 3 Philips Healthcare, Cincinnati, USA

**Keywords:** Steatotic liver disease, Quantitative magnetic resonance imaging, T1 mapping, Proton density fat fraction (PDFF)

## Abstract

**Purpose:**

To evaluate the influence of hepatic fat content and sequence acquisition parameters on Modified Look-Locker Inversion Recovery (MOLLI)-based liver T1 estimates, comparing balanced steady-state free precession (bSSFP) and spoiled gradient recalled echo (SPGR) readouts under in-phase (IP), opposed-phase (OP), and fat-suppressed (FS) conditions.

**Methods:**

Twenty participants (5 controls, 11 with hepatic steatosis, 4 with other chronic liver diseases; median age 15 years, range 10–28) underwent abdominal MRI at 1.5T. Six single-slice MOLLI acquisitions (bSSFP and SPGR; IP, OP, FS) were performed to obtain hepatic T1 maps. A 3D multi-echo confounder-corrected chemical shift encoded (mDIXON Quant^®^) sequence was performed to obtain proton density fat fraction (PDFF), and T2* maps. ROI-based measurements were made by three blinded readers. Agreement and mean difference among MOLLI protocols were assessed using intraclass correlation coefficients (ICC), Bland-Altman analysis, and repeated-measures ANOVA. Correlations between PDFF and T1 differences were evaluated using weighted linear regression. Numerical simulations were implemented to model fat-related effects on MOLLI T1 estimates.

**Results:**

Liver PDFF ranged from 1 to 43% (median 4.6%). Agreement between bSSFP and SPGR T1 estimates was excellent (ICC ≥ 0.90), though SPGR estimated higher T1 values than bSSFP by 30–60 ms, with greater variability. T1-OP differed significantly from T1-IP and T1-FS (both *p* < 0.0001) and showed significant PDFF-related mean difference, in line with simulation results, while T1-FS and T1-IP estimates showed excellent agreement (ICC ≥ 0.89). Regression analysis showed that bSSFP T1-OP correlated very strongly with PDFF (*r* = 0.81; +7.8 ms/%PDFF), whereas T1-IP showed a strong negative correlation (*r* = − 0.79; − 5.0 ms/%PDFF), and T1-FS demonstrated only a moderate negative correlation (*r*=-0.48; -3.1ms/%PDFF). SPGR based T1 estimates exhibited similar correlations with PDFF. In participants without steatosis, T1 values were consistent across all acquisitions.

**Conclusion:**

Liver MOLLI T1 estimates are strongly influenced by hepatic fat content and acquisition parameters, particularly for OP acquisitions. IP and FS MOLLI acquisitions provide more reliable estimates across fat fractions. These findings support prioritization of IP or FS strategies in clinical and research liver T1 mapping protocols and underscore the need for fat-corrected or water-specific approaches.

## Introduction

Hepatic longitudinal relaxation time (T1) has been shown to correlate with fibrosis and inflammation and is being recognized as a potential noninvasive biomarker in assessment of chronic liver disease [1, 2]. Pathological changes in tissue composition directly influence T1 values: intracellular and extracellular edema tend to prolong T1, while fat and iron deposition shorten it [3–7]. Histologic studies have confirmed these opposing effects, with hepatocellular carcinoma (HCC) containing steatosis exhibiting shorter T1 than HCC without steatosis, whereas HCC with fibrosis demonstrates longer T1 [7]. More recent work has shown that hepatic fat can cause either shortening or prolongation of T1 depending on the acquisition sequence [8–10]. Because both tissue architecture and water–fat composition evolve concurrently with liver disease progression, understanding the sequence-specific influence of fat content on T1 estimation is essential, particularly in patients with elevated hepatic fat content such as those with metabolic dysfunction-associated fatty liver disease.

The Modified Look-Locker Inversion Recovery (MOLLI) [11] sequence is widely adopted for clinical T1 mapping due to its efficiency and availability in clinical settings, yet T1 estimates remain sensitive to tissue fat content. Even small amounts of fat can introduce systematic bias [12], with hepatic proton density fat fraction (PDFF) shown to negatively correlate with T1 on in-phase acquisitions and positively on opposed-phase acquisitions, while water-only and water-fat separated approaches reduce influence of fat on T1 estimates [10, 13].

There remains a gap in the literature concerning systematic evaluation of sensitivity of MOLLI-based hepatic T1 estimates to acquisition parameters and hepatic fat content. In this study, we systematically evaluate the influence of hepatic fat content and MOLLI acquisition parameters on hepatic T1 estimates. Specifically, we compare balanced stead-state free precession (bSSFP) and spoiled gradient recalled echo (SPGR) readouts under in-phase (IP), opposed-phase (OP), and fat-suppressed (FS) conditions, to quantify fat-related differences in T1 estimates and identify acquisition strategies less sensitive to fat. We hypothesize that these quantitative evaluations will guide the optimization and harmonization of liver T1 mapping protocols in clinical settings, enhancing diagnostic utility and supporting consistency across multi-center studies.

## Methods

This HIPAA-compliant study was approved by the Institutional Review Board (IRB ID 2023 − 0428). Written informed consent was obtained from participants aged 18 years and older; for minors, parental consent and participant assent (ages 11–17) were obtained.

### Study participants

The study aimed to include approximately one-third of participants with a prior diagnosis of metabolic dysfunction-associated steatotic liver disease (MASLD) or metabolic dysfunction-associated steatohepatitis (MASH), one-third with other chronic liver diseases, and one-third as controls without known liver disease. Recruitment criteria for the study included age between 8 and 80 years, the ability to lie still in the MRI scanner and perform breath-holds of at least 15 s and having no contraindications to MRI. Patients with liver disease were identified and recruited using previous patient diagnoses listed in the Department of Radiology’s electronic medical records. Asymptomatic individuals without known liver disease were recruited through hospital-wide emails and advertisements. Participants were reassigned to the hepatic steatosis subgroup if their measured PDFF was ≥ 6% [14]. This same cohort has been previously reported but looked into influence of fat content on the hepatic T2 estimated using a fast multi-echo 2D Gradient and Spin Echo (GRASE) sequence [15, 16] , and looked into comparing breath-hold and respiratory gated hepatic PDFF, T2* and T2 [15, 16]

### MR image acquisition

All participants were scanned on a 1.5T clinical MRI scanner (Ingenia, Philips Healthcare; Best, the Netherlands) using a torso coil with a combination of 16 anterior and 12 posterior elements. The quantitative imaging protocol included six single slice 2D MOLLI acquisitions for T1 mapping through the mid-liver, and a 3D six-echo chemical shift encoded (mDIXON Quant^®^) acquisition for simultaneous PDFF and T2* mapping of the entire liver. Three MOLLI scans used bSSFP acquisition with (1) echo times (TE) near water and fat in-phase, using shortest available first TE (T1-IP), (2) near opposed-phase, while avoiding banding artifacts (T1-OP) and (3) with spectral inversion recovery fat suppression (T1-FS). The other three MOLLI scans used SPGR acquisitions with matching echo times. All MOLLI scans were acquired during an 11-second end-expiration breath-hold, using a 4s(1s)3s(1s)2 s sampling scheme triggered by a simulated ECG signal providing a trigger every 60 s. The acquisition slice for all MOLLI scans was prescribed at the widest part of the mid-liver in the transverse plane. The central part of the transverse slab for 3D mDIXON Quant^®^ was positioned at the same location. The acquisition parameters for MOLLI scans are detailed in Table 1. For the 3D mDIXON Quant^®^ acquisition, six gradient echoes (with the first TE = 1.2 ms and an echo spacing of 0.8 ms) were acquired using a flip angle of 5°, repetition time (TR) of 6.5 ms. The acquisition covered 24 cm in the caudocephalad direction with an in-plane resolution = 3 × 3 mm^2^, slice thickness 6 mm, and a sensitivity encoding (SENSE) acceleration factor (R) = 2. The breath-hold duration for 3D mDIXON acquisition was 7 s. The PDFF, T2*, and T1 maps were generated on the scanner by FDA-approved vendor software.

**Table 1:** Imaging parameters for 2D modified Look Locker inversion recovery (MOLLI) acquisitions

	MOLLI bSSFP			MOLLI SPGR		
	IP	OP	FS	IP	OP	FS
FOV (mm)	440 × 340	440 × 340	440 × 340	440 × 340	440 × 340	440 × 340
Slice thickness (mm)	10	10	10	10	10	10
Resolution (mm)	3 × 3	3 × 3	3 × 3	3 × 3	3 × 3	3 × 3
CS-SENSE	2.3	2.3	2.3	2.3	2.3	2.3
Flip angle (º)	35	35	35	5	5	5
TR (ms)	1.9	4.8	1.97	1.9	4.8	1.97
TE (ms)	0.59	1.93	0.91	0.59	1.93	0.91
Startup echoes	10	10	10	4	4	4
Partial Fourier factor	1	0.75	0.7	1	0.75	0.7
Turbo factor	50	40	37	50	40	37
Fat sat	–	–	SPIR	–	–	SPIR

*bSSFP* balanced steady-state free precession; *FS* fat suppressed; *IP* water and fat in-phase; *OP* water and fat opposed-phase; *SPGR* spoiled gradient-echo; *FOV* field of view; *CS-SENSE* compressed-sensing sensitivity encoding; *TR* repetition time; *TE* echo time; *SPIR* spectral inversion recovery

### Image analysis

Anatomically matched PDFF, T2*, and T1 measurements were independently performed by three blinded readers: a pediatric radiology research fellow, a Department of Radiology research assistant, and an MRI clinical scientist (PD, AKR, JSG). The image quality of all acquisitions, including the generated PDFF, T2*, and T1 maps, was visually evaluated for motion artifacts and noise within the liver. Each reader placed three circular regions of interest (ROIs > 100 mm^2^) in the right liver (anterior, lateral, and posterior) and one in the paraspinal muscle, on all T1 maps and the corresponding slice from PDFF and T2* maps. ROIs were anatomically matched across sequences, with minimal spatial adjustments allowed to avoid areas < 95% confidence on the scanner-generated quantitative maps, as well as the liver capsule, parenchymal edges, visible blood vessels, bile ducts, and any visible artifacts. All ROI placements were reviewed by a board-certified pediatric radiologist with more than ten years of post-fellowship experience (ATT). The mean and standard deviation of the values within each ROI were recorded.

### Numerical simulations

Numerical Bloch equation [17] simulations were performed in MATLAB (The MathWorks™ Inc., Natick, Massachusetts, USA) to model the influence of hepatic fat content on MOLLI-derived T1 estimates. Tissue properties were defined at 1.5T with relaxation times representative of liver (T1 = 500 ms, T2 = 50 ms) and fat (T1 = 280 ms, T2 = 60 ms), incorporating a single-peak model with 3.5 ppm chemical shift between water and the dominant methylene fat peak [1–3]. Water and fat proportions were varied from 0 to 50% fat in 1% increments. Simulations incorporated single-echo SPGR and bSSFP MOLLI acquisitions using a 4s(1s)3s(1s)2 s sampling scheme and employed a three-parameter exponential recovery fit with conditional data inclusion followed by a Look-Locker correction [18]. Sequence parameters—including flip angle, start-up echoes, partial Fourier factors, and in-phase/opposed-phase TE and associated TR—were matched to the acquisition settings used in research participants. Additional modeling accounted for vendor-implemented RF flip angle ramp-up, phase cycling, and incorporated spin-frequency distribution spanning 1.5T ± 3 ppm to reflect realistic off-resonance effects [19].

### Statistical analysis

Descriptive statistics were used to summarize demographics. The normality of the continuous variables was assessed using Shapiro-Wilk test. Continuous variables were reported as either mean ± standard deviation (SD) or as median with interquartile range (IQR), as appropriate, while categorical variables were presented as counts. Inter-reader agreement for measurements was assessed using intraclass correlation coefficient (ICC). Measured values were averaged across readers per ROI. Correlation and agreement of estimated T1s among MOLLI protocols were assessed using Pearson’s correlation (r), intra-class correlation coefficients (ICC), and Bland-Altman analysis.

Statistical comparisons of T1s across acquisition protocols were performed using repeated measures ANOVA to assess overall protocol effects, with Bonferroni-corrected post hoc pairwise tests applied for multiple comparisons. Group-level distributions of T1s were visualized using box-and-whisker plots overlaid with individual ROI values. Relationships between PDFF and difference in T1 estimates were assessed using weighted linear regression with inverse density-based weights to account for non-uniform data distribution.

A p-value of less than 0.05 was considered statistically significant for all inference testing, and 95% confidence intervals were calculated as appropriate. ICC values were interpreted as follows: < 0.5, poor; 0.5–0.75, moderate; 0.75–0.9, good; and > 0.90, excellent agreement [20]. Correlation coefficients were interpreted as follows:0–0.19, very weak; 0.2–0.39, weak; 0.40–0.59, moderate; 0.60–0.79, strong; and 0.80–1.0, very strong [21]. All statistical analyses were performed using MATLAB (The MathWorks™ Inc., Natick, Massachusetts, USA).

## Results

Twenty participants (9 males, 11 females; mean age 16.1 years ± 5.4 [range: 10–28 years]) successfully underwent liver MR imaging for research purposes. Baseline diagnoses included: 7 with MASLD, 3 with MASH, 4 with other non-steatotic chronic liver diseases, and 6 asymptomatic volunteers with no known liver disease. Table 2 summarizes participant characteristics at the time of recruitment.

**Table 2:** Characteristics of the study population

Number of patients	20
Age (year)	16.1 ± 5.4 (10–28)
Female-to-male ratio	11:9
Height (cm)	159.3 ± 16.6 (128–193)
Weight (kg)	72.2 ± 27.3 (34–135)
BSA (m^2^)	1.76 ± 0.39 (1.1–2.5)
BMI (kg/m^2^)	28.1 ± 8.8 (18.2–50.4)
Clinical Indications	
Asymptomatic	6
MASLD	7
MASH	3
Sclerosing cholangitis	1
Chronic hepatitis B	1
Autoimmune hepatitis	1
Congenital hepatic fibrosis	1

**Fig. 1:**
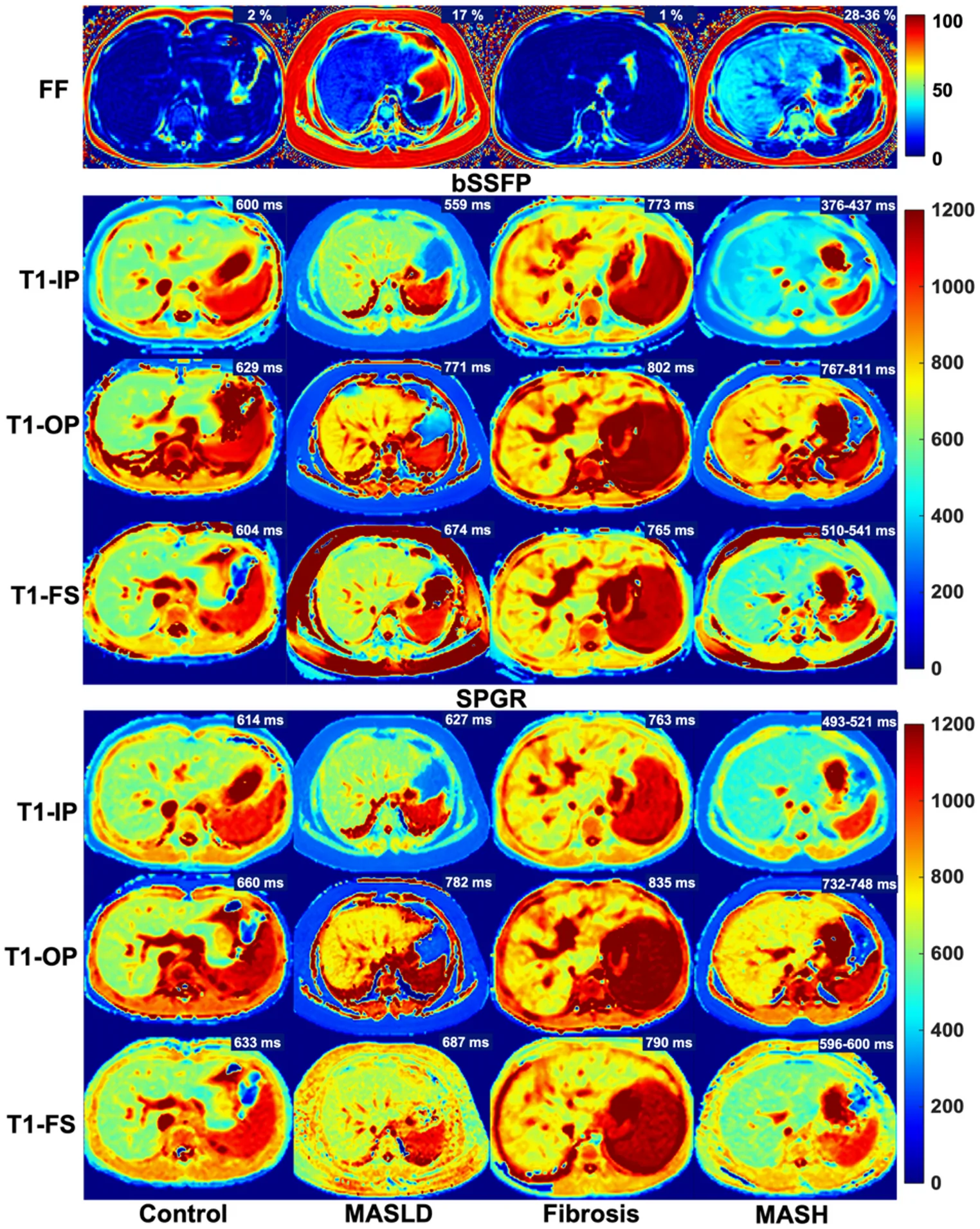
Representative maps from four participants (columns) showing proton density fat fraction (PDFF) from mDIXON Quant^®^ and T1 values obtained using MOLLI acquisitions with bSSFP (top) and SPGR (bottom) readouts. The top row displays PDFF maps from 3D six-echo mDIXON Quant^®^. The second, third, and fourth rows show T1 maps from MOLLI acquisitions using IP echo times, OP echo times, and FS, respectively. Numbers in top right show the mean of the three reader-averaged ROI values, or the range when the standard deviation of PDFF across the nine ROI measurements (three ROIs × three readers) exceeded 2%. Participants (left to right): control (15-year-old female); biopsy-confirmed MASLD/borderline MASH (10-year-old male); sclerosing cholangitis with advanced fibrosis (19-year-old male); and elevated liver enzymes with ultrasound shear wave speed of 1.9 m/s and clinical diagnosis of MASH (11-year-old male)

The quality of acquired images and generated quantitative maps was deemed adequate for ROI placement by both a pediatric radiology research fellow (PD) and a board-certified radiologist (ATT). Inter-reader agreement was good to excellent for PDFF (ICC > 0.99), T2* (ICC > 0.87) and all six T1 estimates (ICC > 0.94) across the three readers. Mean PDFF, T2* and T1 values across three readers were used for further analysis. Figure 1 presents PDFF and MOLLI T1 maps for four representative participants. In control participants and participants with fibrotic disease, hepatic T1 values remained comparatively lower and higher, respectively, regardless of echo time or fat suppression. In contrast, hepatic T1-OP values were elevated compared to T1-IP and T1-FS for both bSSFP and SPGR readouts in the participants with MASLD and MASH.

Across participants, liver PDFF ranged from 1% to 43% (median 4.6%, IQR 28%) and T2* from 9 ms – 36 ms (median 25.8 ms, IQR 5.2 ms). Notably, one participant with a prior diagnosis of primary sclerosing cholangitis and autoimmune hepatitis exhibited an outlier low T2* value of 9 ms. Based on the PDFF ≥ 6% threshold, one asymptomatic volunteer was reclassified as steatotic, while two participants with history of MASLD but current PDFF < 6% were reclassified as non-steatotic. Final groups were: 7 non-steatotic liver (NSL), 4 non-steatotic liver disease (NSLD), and 9 steatotic liver disease (SLD). In participants without hepatic steatosis (NSL and NSLD, liver PDFF ranged from 1% to 5% (2.9 ± 1.2%) and muscle PDFF ranged from 2% to 7% (4.2 ± 1.8%). In SLD participants, liver PDFF ranged from 8% to 43% (28.8 ± 11.9%) and muscle PDFF ranged from 4% to 15% (median 6.1%, IQR 5.2%).

Summary of correlations and agreements between each of the six MOLLI acquisition based T1 values of liver and paraspinal muscle across all participants is provided in Table 3. Agreement between bSSFP and SPGR MOLLI T1 values was excellent (T1-IP ICC = 0.94 [95% CI: 0.91–0.96], T1-OP ICC = 0.93 [95% CI: 0.89–0.95]), T1-FS ICC = 0.90 [95% CI: 0.84–0.93]). SPGR consistently estimated higher T1 values than bSSFP (T1-IP −57.15 [−146.00, 31.71] ms, T1-OP −28.19 [−131.01, 74.64] ms, T1-FS −58.98 [−156.86, 38.90] ms). Repeated measures ANOVA revealed significant (*p* < 0.0001) difference among the T1 values from IP, OP, and FS sequences for both bSSFP and SPGR acquisitions. Post-hoc comparisons showed T1-OP differed significantly from both T1-IP and T1-FS (*p* < 0.0001, *p* < 0.0001), while T1-IP and T1-FS did not differ significantly (*p* = 0.43, *p* = 0.35) for both bSSFP and SPGR acquisitions. T1-OP consistently yielded higher values and greater variability compared to T1-IP and T1-FS for both bSSFP and SPGR, reflected in lower ICCs and wider limits of agreement. In contrast, T1-IP and T1-FS demonstrated stronger consistency and smaller mean difference for both bSSFP (7.1 [−99.2, 113.4] ms) and SPGR (2.93 [−82.2, 88.1] ms).

**Table 3:** Summary table of correlation and agreement for All T1 estimates

Comparison	Pearson *r* [95% CI]	ICC [95% CI]	Mean difference [LoA] (ms)
bSSFP			
T1-IP vs. T1-OP	0.13 [−0.09, 0.34]	0.13 [−0.09, 0.34]	−149.41 [−501.50, 202.69]
T1-IP vs. T1-FS	0.91 [0.86, 0.94]	0.89 [0.83, 0.93]	−29.59 [−143.09, 83.92]
T1-OP vs. T1-FS	0.32 [0.11, 0.51]	0.31 [0.10, 0.49]	119.82 [−164.98, 404.62]
SPGR			
T1-IP vs. T1-OP	0.64 [0.49, 0.75]	0.64 [0.49, 0.75]	−120.44 [−340.25, 99.36]
T1-IP vs. T1-FS	0.94 [0.90, 0.96]	0.93 [0.89, 0.95]	−31.42 [−123.47, 60.62]
T1-OP vs. T1-FS	0.81 [0.72, 0.87]	0.80 [0.70, 0.87]	89.02 [−64.13, 242.17]
bSSFP vs. SPGR			
T1-IP vs. T1-IP	0.94 [0.91, 0.96]	0.94 [0.91, 0.96]	−57.15 [−146.00, 31.71]
T1-OP vs. T1-OP	0.93 [0.89, 0.95]	0.93 [0.89, 0.95]	−28.19 [−131.01, 74.64]
T1-FS vs. T1-FS	0.90 [0.85, 0.93]	0.90 [0.84, 0.93]	−58.98 [−156.86, 38.90]

*ICC* intraclass correlation coefficient; *LoA* limits of agreement; *T1-IP, T1-OP, T1-FS - T1* estimates from MOLLI acquisitions using IP (in-phase) echo times, OP (opposed-phase) echo times, FS (fat suppression); *bSSFP* balanced steady-state free precession; *SPGR* spoiled gradient-echo

**Fig. 2:**
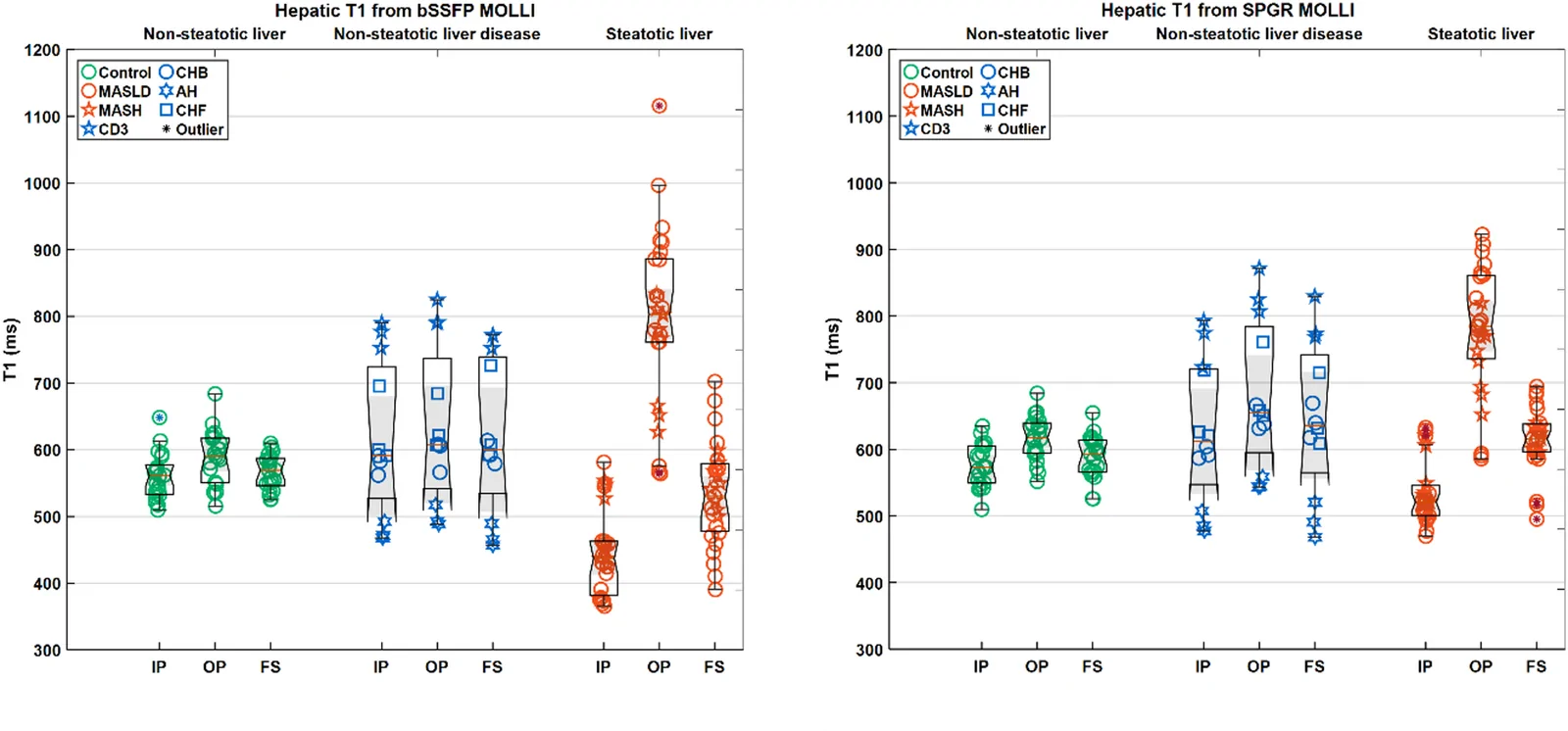
Tukey box plots of hepatic T1 values obtained from bSSFP (left) and SPGR (right) MOLLI acquisitions. T1-IP, T1-OP, and T1-FS values from three liver ROIs are shown for three participant groups: non-steatotic liver (*n* = 7), non-steatotic liver disease (*n* = 4), and steatotic liver disease (*n* = 9). Each box represents the interquartile range (IQR), with the center red line indicating the median. Whiskers extend to the minimum and maximum values within 1.5× IQR. Non-overlapping notches suggest a statistically significant difference in medians at the 5% level. CD3 = CD3 T cell-mediated scleorsing cholangitis with advanced liver fibrosis, CHB = Chronic hepatitis B, AH = Primary sclerosing cholangitis and autoimmune hepatitis, CHF = Congenital hepatic fibrosis

**Fig. 3:**
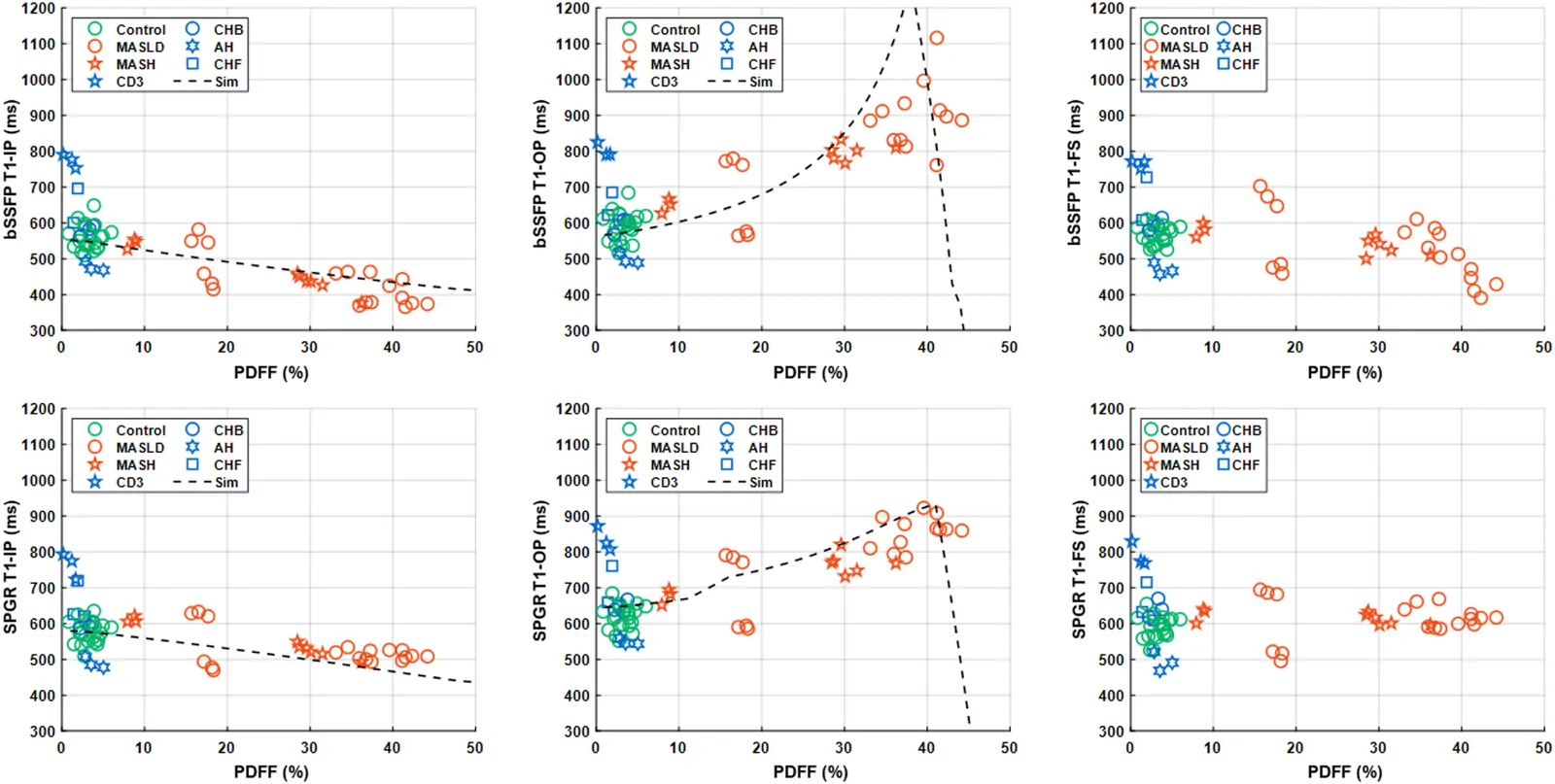
Hepatic T1 values obtained from bSSFP (top row) and SPGR (bottom row) MOLLI acquisitions for IP (left column), OP (center column), and FS (right column) configurations, plotted against PDFF values measured from mDIXON Quant^(R)^. The plots demonstrate consistent associations between PDFF and MOLLI-derived hepatic T1 values, with opposite directional trends observed for IP and OP acquisitions across both bSSFP and SPGR readouts. In contrast, T1-FS values are relatively insensitive to PDFF. Simulated trends (dashed lines) were generated using tissue properties at 1.5T with representative relaxation times for liver (T1 = 500 ms, T2 = 50 ms) and fat (T1 = 280 ms, T2 = 60 ms), illustrating how changes in water–fat proportion alone influence estimated T1 values for each MOLLI acquisition. CD3 = CD3 T cell-mediated scleorsing cholangitis with advanced liver fibrosis, CHB = Chronic hepatitis B, AH = Primary sclerosing cholangitis and autoimmune hepatitis, CHF = Congenital hepatic fibrosis

**Fig. 4:**
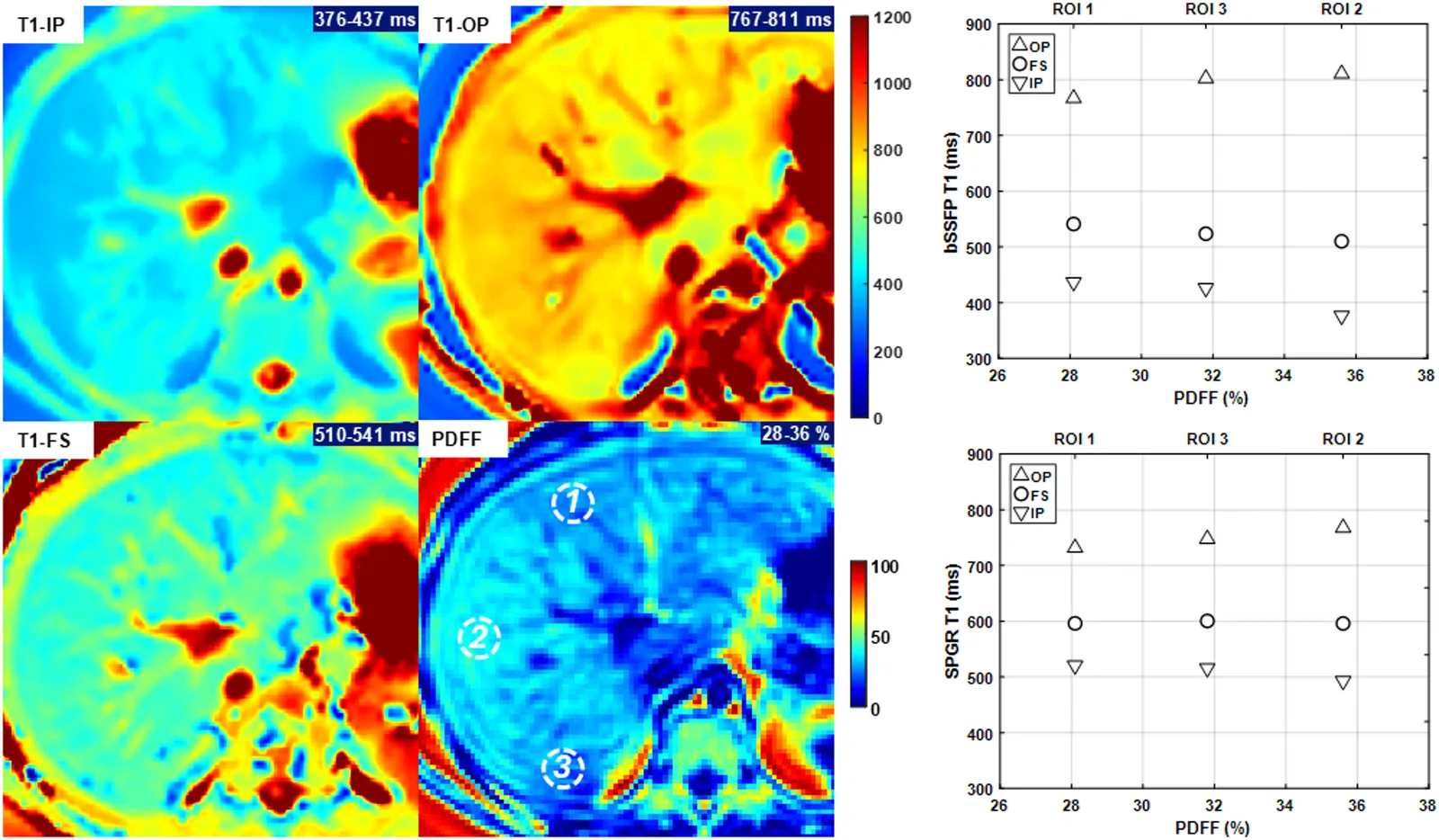
An 11-year-old male with elevated liver enzymes, ultrasound shear wave speed of 1.9 m/s, and clinical diagnosis of MASH demonstrated heterogeneous hepatic fat distribution, with PDFF ranging from 28% to 36% across three liver ROIs overlaid on the PDFF map. T1-IP, T1-OP, and T1-FS maps are shown from bSSFP MOLLI acquisitions. Corresponding bSSFP and SPGR T1 values acquired using IP, OP, and FS MOLLI acquisitions are shown in the adjacent scatter plots. T1-OP values increased with PDFF, while T1-IP decreased, and T1-FS remained relatively stable across ROIs. The marked difference in T1-OP between the right lateral (ROI 2) and right anterior (ROI 1) liver segments reflects the TE-dependent influence of fat on MOLLI-based T1 estimation

Group-wise distributions of T1 values from all acquisitions across participant subgroups confirmed these trends while highlighting the pronounced divergence of hepatic T1-OP values from T1-IP and T1-FS acquisitions in the SLD subgroup compared to other subgroups for both bSSFP and SPGR (Fig. 2). Similarly, paraspinal muscle T1-OP differed significantly from T1-IP and T1-FS (*p* < 0.0001) for both the bSSFP and SPGR acquisitions. Numerical simulations demonstrated that hepatic T1 estimates increased with rising PDFF for OP echo times and decreased with rising PDFF for IP echo times for both bSSFP and SPGR readouts. Figure 3 shows simulation results of T1 values as a function of PDFF overlaid with the participant T1 estimates. Correlation and weighted linear regression analyses demonstrated significant associations between PDFF and MOLLI-derived hepatic T1 values, with opposite trends for in‑phase and opposed‑phase acquisitions across both bSSFP and SPGR readouts, in line with the simulation results. For bSSFP, T1-IP showed a strong negative correlation with PDFF (*r* = −0.79, slope − 5.0 ms/%PDFF, R² = 0.75, *p* < 0.0001), while T1-OP demonstrated a very strong positive correlation (*r* = 0.81, slope + 7.8 ms/%PDFF, R² = 0.72, *p* < 0.0001). T1-FS exhibited only a moderate negative correlation (*r* = −0.48, slope − 3.1 ms/%PDFF, R² = 0.34, *p* < 0.0001). Similarly, for SPGR acquisitions, T1-IP correlated moderately and negatively with PDFF (*r* = −0.55, slope − 2.6 ms/%PDFF, R² = 0.44, *p* < 0.0001), whereas T1-OP correlated strongly and positively (*r* = 0.75, slope + 5.6 ms/%PDFF, R² = 0.67, *p* < 0.0001). In contrast, T1-FS showed no significant association with PDFF (*r* = −0.004, slope − 0.1 ms/%PDFF, R² = 0.00, *p* = 0.84). Figure 4 shows a participant with heterogeneous PDFF that highlights how hepatic fat content can introduce differences in MOLLI T1 estimates, particularly for OP acquisitions, and underscores the relative robustness of IP and FS strategies in heterogeneous livers.

**Fig. 5:**
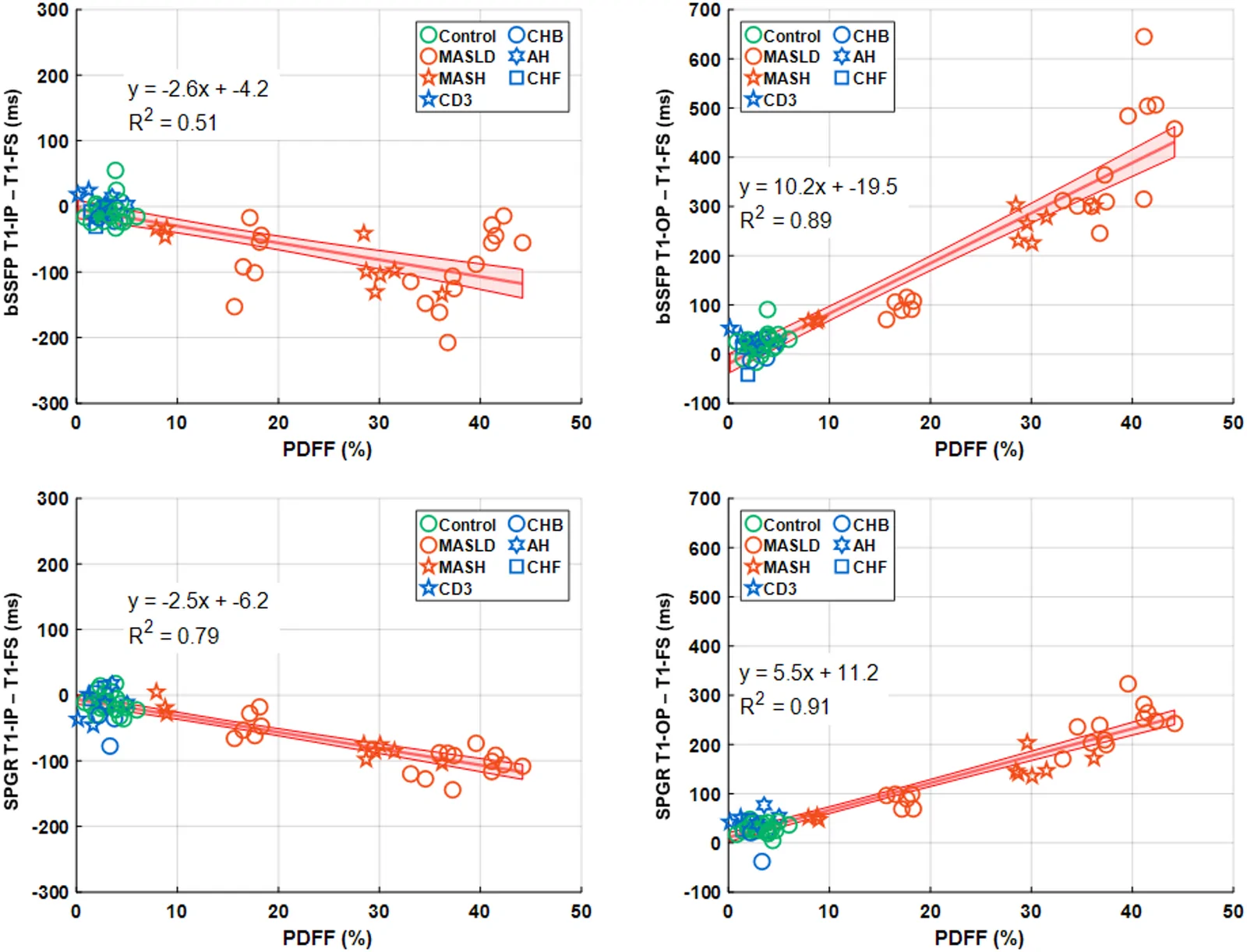
Differences in hepatic T1-IP (left column) and T1-OP (right column) values relative to corresponding T1-FS values for bSSFP (top row) and SPGR (bottom row) MOLLI acquisitions, plotted against PDFF values measured from mDIXONQuant. The plots demonstrate consistent associations between PDFF and MOLLI-derived hepatic T1 values, with opposite directional trends observed for IP and OP acquisitions across both bSSFP and SPGR readouts. CD3 = CD3 T cell-mediated scleorsing cholangitis with advanced liver fibrosis, CHB = Chronic hepatitis B, AH = Primary sclerosing cholangitis and autoimmune hepatitis, CHF = Congenital hepatic fibrosis

Weighted regression analyses demonstrated significant associations between liver PDFF and differences in hepatic T1-IP and T1-OP with respect to corresponding T1-FS for both bSSFP and SPGR MOLLI acquisitions (Fig. 5). Across participants, T1-OP – T1-FS differences showed very strong positive correlations with PDFF (*r* = 0.94 for bSSFP, 0.96 for SPGR), whereas T1-IP – T1-FS differences demonstrated moderate negative correlations (*r* = − 0.72 for bSSFP, 0.89 for SPGR). Overall, the regression analyses reinforce the subgroup findings that hepatic T1-OP consistently diverged from T1-IP and T1-FS with increasing PDFF, whereas T1-IP and T1-FS remained closely aligned with negligible mean difference. These results highlight the robustness of IP and FS acquisitions across fat fractions and the susceptibility of OP acquisitions to PDFF-driven deviation.

## Discussion

This study systematically evaluated the influence of hepatic fat content on MOLLI-derived T1 estimates across bSSFP and SPGR readouts with IP, OP, and FS acquisitions. Hepatic T1 estimates were strongly influenced by fat fraction and acquisition strategy: T1-OP estimates consistently diverged from T1-IP and T1-FS with increasing PDFF, while T1-IP and T1-FS estimates remained closely aligned with negligible mean difference. Across participants, T1-OP and T1-FS differences showed strong positive correlations with PDFF, whereas T1-IP and T1-FS differences demonstrated moderate negative correlations. Agreement between bSSFP and SPGR sequences was excellent (ICC > 0.90), though SPGR consistently estimated higher T1 values than bSSFP by 30–60 ms.

In addition to group-level trends, our study highlights the influence of spatially heterogeneous fat distribution in diffuse liver disease on T1 estimates. In one participant with MASH, PDFF varied from 24.8% to 33.1% across ROIs, with T1-OP prolonged and T1-IP shortened relative to T1-FS. Notably, the marked difference in T1-OP between the right lateral (ROI 2) and right anterior (ROI 1) portions of the liver was driven by the TE-dependent influence of fat on MOLLI-based T1 estimation. This case illustrates how regional PDFF variability can introduce differences in MOLLI T1 estimates, particularly for OP acquisitions, and underscores the robustness of IP and FS strategies in heterogeneous livers. Importantly, within the subgroup of participants with non-steatotic liver disease, hepatic T1 values remained consistent across IP, OP, and FS acquisitions for both bSSFP and SPGR. This included the participant with the outlier low T2* (9 ms, PDFF 3%), who showed SPGR T1 values of 480–550 ms and bSSFP T1 values of 470–520 ms across IP, OP, and FS acquisitions over three ROIs. These findings suggest that, in this case, the low T2* did not meaningfully interfere with MOLLI-derived T1 measurements This stability reinforces that fat-driven deviation is the primary driver of sequence-dependent variability, and that in the absence of steatosis, MOLLI T1 estimates are reproducible across acquisition strategies.

Clinically, these findings suggest that OP acquisitions should be avoided when reproducibility is critical, as they are highly susceptible to fat-driven deviation and may exaggerate T1 elevations in patients with steatotic liver disease, potentially obscuring fibrosis- or inflammation-related changes. In contrast, IP and FS acquisitions provide more reliable estimates across fat fractions, with narrow limits of agreement to within ± 90 ms. These results support prioritization of IP or FS MOLLI acquisitions in clinical and research protocols, particularly in populations with elevated hepatic fat.

While IP and FS acquisitions remain robust, their implementation carries important technical constraints. Fat suppression reduces fat-driven deviation but at the cost of lower signal-to-noise ratio (SNR), which may limit precision in advanced disease or multi-slice imaging. Acquiring true in-phase TE requires prolongation of the TR, constraining excitations per readout and limiting spatial resolution; this trade-off must be balanced against preserving T1 recovery. In bSSFP, the shortest possible TE should be used to minimize banding artifacts, necessitating IP acquisition at the shortest TE available, as close as possible to true in-phase timing in both bSSFP and SPGR. Finally, bSSFP inherently provides higher SNR than SPGR, which may explain its tighter limits of agreement and stronger reproducibility. Taken together, these constraints highlight that while IP and FS acquisitions mitigate fat-driven deviation, their implementation requires careful optimization of TE, TR, and suppression strategies to balance SNR, spatial resolution, and artifact reduction.

Moreover, fat-driven deviation extends beyond native T1 measurements to derived metrics such as extracellular volume (ECV), reinforcing the need to account for PDFF in chronic liver disease. Truly water-specific T1 estimation methods—such as water-only or water–fat separated approaches—are ultimately desirable, as they directly eliminate fat-related confounding [10, 13, 22]. Until such techniques are clinically validated and broadly available, careful optimization of MOLLI acquisition parameters is essential to ensure reproducibility, minimize fat-driven deviation, and support reliable biomarker development.

Several limitations should be acknowledged. The sample size was modest and drawn primarily from adolescents at a single pediatric center, which limits statistical power and may limit generalizability to adult populations and the broader spectrum of chronic liver disease. Imaging was performed at 1.5T; results may differ at 3 T where chemical shift and off-resonance effects are more pronounced. We focused on single-slice MOLLI acquisitions, whereas volumetric mapping may introduce additional variability. Finally, histologic correlation was not available, precluding direct assessment of diagnostic accuracy for fibrosis or inflammation.

Future studies should expand these findings in larger, multi-center cohorts to validate reproducibility across diverse populations and scanner platforms. Performing analogous evaluations at 3 T will be essential to assess how fat-driven deviations in T1 estimation behave at higher field strengths. Volumetric T1 mapping and automated ROI placement strategies could further improve reproducibility and reduce operator dependence. Integration of water–fat separation techniques or fat-correction algorithms into MOLLI reconstruction may enhance accuracy without requiring additional acquisitions. Finally, coupling quantitative T1 mapping with histologic correlation for fibrosis and inflammation will be critical to establish diagnostic thresholds and support biomarker qualification for clinical trials and routine practice.

## Conclusion

Hepatic MOLLI-derived T1 estimates are strongly influenced by fat fraction and acquisition strategy. T1 estimates from OP sequences consistently diverged from IP and FS acquisitions with increasing PDFF, attributable to systematic overestimation of T1-OP estimates, while T1-IP and T1-FS remained robust with negligible mean difference. Although bSSFP and SPGR-based T1 estimates showed excellent agreement, SPGR consistently estimated higher T1 values than bSSFP, underscoring the need for harmonization. Technical constraints—including SNR penalties with fat suppression, TR prolongation for true in-phase echoes, and TE limitations to avoid banding—must be balanced when optimizing protocols. Until truly water-specific T1 mapping becomes clinically validated and widely available, careful attention to MOLLI acquisition parameters is essential to ensure reproducibility and reliability in chronic liver disease.

## Data Availability

The anonymized data underlying this study may be made available upon reasonable request to the corresponding author, subject to IRB approval and compliance with applicable privacy regulations.
